# Elderly and bladder cancer: The role of radical cystectomy and orthotopic urinary diversion

**DOI:** 10.1177/03915603241240644

**Published:** 2024-03-29

**Authors:** Francesco Pio Bizzarri, Eros Scarciglia, Pierluigi Russo, Filippo Marino, Simona Presutti, Seyed Koosha Moosavi, Mauro Ragonese, Marco Campetella, Carlo Gandi, Angelo Totaro, Giuseppe Palermo, Emilio Sacco, Marco Racioppi

**Affiliations:** 1 Department of Urology Fondazione Policlinico “Agostino Gemelli”, Università Cattolica del Sacro Cuore, Roma, Italy; 2 Urology Department, Isola Tiberina-Gemelli Isola Hospital, Catholic University Medical School, Rome, Italy

**Keywords:** Bladder cancer, elderly population, muscle invasive bladder cancer, orthotopic neo-bladder, urinary diversion

## Abstract

The incidence of bladder cancer (BC) depends on advancing age and other risk factors, significantly impacting on surgical, functional and oncological outcomes. Radical cystectomy (RC) with urinary diversion is the gold standard therapy for muscle invasive bladder cancer; however, it remains a complex surgery and requires careful analysis of risk factors in order to potentially decrease post-surgical complication rates. Age in surgery is a limiting factor that can modify surgical and oncological outcomes, and is correlated with a high rate of post-dimssion hospital readmissions. The reconstruction of the bladder with the intestine represents a crucial point of radical cystectomy and the urinary derivation (UD) is at the center of many debates. A non-continent UD seems to be the best choice in elderly patients (>75 years old), while orthotopic neobladder (ON) is poorly practiced. We reviewed the literature to identify studies reporting outcomes, complications, patient- selection criteria, and quality-of-life data on elderly patients, who underwent ON following radical cystectomy. Reviewing the literature there is no clear evidence on the use of age as an exclusion criterion. Certainly, the elderly patient with multiple comorbidities is not eligible for ON, preferring other UD or rescue therapies. A careful preoperative selection of elderly patients could greatly improve clinical, surgical and oncological outcomes, giving the chance to selected patients to receive an ON.

## Introduction

The incidence of bladder cancer (BC) depends on advanced age and other risk factors, which also have a significant impact on surgical, functional and oncological outcomes. Radical cystectomy (RC) is the therapy of choice for muscle invasive bladder cancer (MIBC) and there are several debates in literature on the type of urinary diversion to perform. However, it remains a complex surgery and requires careful analysis of risk factors in order to potentially reduce post-surgical complication rates.

Nowadays BC care is a common problem, particularly for the elderly population (>75 years). The mean age of MIBC onset is 73 years and in approximately 30% of cases it occurs in patients over 75 years. The term “elderly” literally refers to advanced chronological age but in a broader sense it includes the functional status of each individual patient. Population aging is becoming a challenging problem for the healthcare system, both in terms of therapeutic choices and clinical management. Due to the numerous comorbidities, therapeutic choices for elderly patients become complex, and in most cases a multidisciplinary approach is required. The progressive aging of the population represents a serious issue for BC, since it is associated to worse oncological and surgical outcomes and to worse stage of disease at diagnosis.^1^ Elderly patients usually present the most aggressive disease at diagnosis and a significant restriction of the therapeutic choices. Far fewer elderly patients undergo radical surgical treatment for MIBC, than young people. In fact, according to literature, the rate of postoperative complications significantly increases in elderly population. The decision to treat or not these patients is not at all simple and it depends on the Quality of life (QoL), too. Furthermore, RC is technically difficult to perform in these patients, for whom other therapeutic options, such as radiation therapy^2^ or trimodal therapy,^3^ could be chosen. In patients younger than 60 years, radical treatment is applied in approximately 60% of new MIBC diagnosis. In those over 80 years, only 12% are treated with radical cystectomy, and among these, many radical surgical treatments have a palliative and not-therapeutic intent.^4^

## Patient selection

In all the studies collected, pre-operative patient selection plays a key role, in order to ensure a rapid post-operative recovery, particularly in the elderly.^5^ In general, elderly patients (as appropriate age >80^6^ or >75^7^) are less frequently subjected to surgery with radical intent, due to their frailty. In a study by Horovitz et al.^8^ involving 605 patients who underwent RC, all the 47 patients older than 80 years received an ileal conduit (IC). Similarly, in a Memorial Sloan Kettering study, none of the 44 patient older than 80 years, who underwent RC from 1980 to 1995, received an orthotopic neobladder (ON).^9^

A study by Schiffmann et al.^10^ analyzed mainly elderly patients who underwent radical cystectomy from 1991 to 2009. In the first decade, elderly patients (⩾80 years) were 20.9% and in the second decade 29.6% of cases; in addition, the percentage of patients with a Charlson Comorbidity index (CCI) score of ⩾3 increased from 19.3 to 30.5% across the two decades and 25.9% of patients were octogenarians. All these data indicate that nowadays more elderly and frail patients are treated with radical cystectomy than in the past, and a wide disparity among urinary diversions selection still exists. Furthermore, a study conducted by Chamie et al.^6^ demonstrated that patients over than 80 years who received a RC had improved cancer specific survival compared with those who did not. A review of 20 studies about RC was performed by Froehner et al.,^11^ analyzing perioperative mortality and complication rates in elderly patients: it turned out that in this group 70% of diversions were incontinent diversions and 30% were continent. In patients with ON the continence rate is lower than in younger patients, with similar oncological outcome after RC. In literature, there are currently no randomized trials comparing the types of urinary shunts after radical cystectomies in elderly patients. However, we can intuitively assume that elderly patients in good health (with low CCI scores) may have better post-operative outcomes, but in literature few data exist to guide the clinicians in decision making. Finally, it is important to have good counseling with the patient, in order to propose the best therapeutic option in the most appropriate way. Indeed, in one study from Ashley and Daneshmand all patients deemed eligible for ON received standardized preoperative counseling. Among these only 9% of patient selected an incontinent diversion.^12^ Frailty represents a very useful prognostic factor in predicting post-operative risks and it is also being considered in RC.^13^^14^ Although several scales are used to define frailty, there is no standardized criteria that can give a univocal definition of “frail patient.” The “frailty phenotype” defined by Fried et al.^15^ includes three or more of the following characteristics: decreased walking time and gait speed, diminished grip strength, decreased physical activity levels, a feeling of exhaustion after activity, and unintentional weight loss in 1 year. Another way to analyze frailty is the “comprehensive geriatric assessment (CGA),” which entirely analyzes the patient, including multidimensional and multidisciplinary aspects, and also provides care plans. It consists in a systematic global examination of the patient across multiple domains to identify specific risk factors, like functional status, comorbidities, cognition, nutritional status and psychological status.^16^

## Survival outcomes

The therapeutic utility of RC in elderly patients has been demonstrated in several studies.^17^ These studies had compared young and elderly patients undergoing RC and only two studies had highlighted the differences between urinary leads in the same age group. However, one study^18^ reported survival outcomes in 85 patients over than 75 years treated with RC, 35 of them received an ON, while 53 patients received an IC as urinary derivation. The follow up was performed in 34 months and there was no difference in terms of overall survival (OS) between the groups (*p* = 0.77).

Clark et al.^19^ analyzed data from 1054 patients who underwent RC from 1971 to 1997. Elderly patients (364) were stratified by either IC or neobladder and no differences in operative mortality between the two groups (*p* = 0.21) were found. Wuethrich et al.^20^ analyzed 244 old patients (age >75 years) who underwent RC with urinary diversion, including ON, IC and ureterocutaneostomy (UCS), during a period of 3 years. The data collected documented that the 90-day complication rate was 54.3% in the ON, 56.7% in the IC group, and 63.6% in the UCS group. Mean estimated OS was 90 months in the ON, 47 months in the IC, and 11 months in the UCS group. Mean estimated CSS was 98 months in the ON, 91 months in the IC, and 12 months in the UCS group.

## Urinary derivation and continence

It must be noticed that the definition of continence and incontinence vary between all studies, making it difficult to have a standardized comparison between different data collected from the mentioned studies. The clinical experience of Sogni et al. has showed that among elderly patients (⩾75 years) the rate of continence (use of 0 pad) was of 56% during the day and 25% during the night.^18^ Ahmadi et al.^21^ conducted a clinical trial between 2002 and 2009 studying continence rates in male with ON. 179 patients with a mean age of 70 years were analyzed about their continence status. In this study 47% of patients used at least one pad during the day, while 72% of patients wore at least one pad at night. Authors observed that advanced age and the presence of systemic pathologies such as diabetes mellitus affects continence but not on the quantity of PADs used on a daily basis. Hugen and Daneshmand reported the day and night-time continence data at a mean of 21 months following surgery in 221 male patients (⩾70 years old) who underwent RC and ON.^22^ Questionnaires were administered to 164 patients and a continence rate of 12% was documented, 33.5% of patients used PADs for protection, 21% were incontinent and used one PAD per day, 33.5% reported severe incontinence with use of two or more PADs per day. Unfortunately, there is not enough data regarding elderly female continence following ON. About this, Todenhöfer et al.^23^ conducted a review including outcomes of seven studies involving 503 women, with a median age ranged from 50 to 69 years, who underwent ON. The review demonstrated that age was associated with nocturnal incontinence. A meta-analysis by Steers^24^ involving 2238 patients who underwent neobladders following RC, showed a mean daytime incontinence rate of 13.3% and mean night-time incontinence rate of 28.5%. According to the above mentioned study, daytime continence after 1 year was achieved in 66% of patients (considered as “dry” by authors), satisfactory (⩽1 pad/day) in 20% of patients, and unsatisfactory (>1 pad/day) in 14% of patients. Night-time continence was achieved in 46% of cases, satisfactory in 26%, and unsatisfactory in 29%.

## Peri-operative complications

RC remains a morbid operation for patients, despite innovations in the surgical field such as the use of laparoscopic and robotic devices. The analysis of modifiable preoperative risk factors is essential to achieve a successful intervention and reduce complication rates. In many cases advanced age (75> yo) has been associated with peri and postoperative complications following RC,^2^ however in literature there are no studies comparing complication rates based on the type of UD. In 2005 a study by Clark et al.^19^ evaluated the complication rates following RC. The patients were divided into four age groups (<60 years, 60–69, 70–79, and > 80 years). Patients older than 70 years have higher complication rates than the others, but focusing only on elderly patients, there are no differences between early complications, early diversion-related complications (like urinary leakage, ureteral stenosis, fistula, uretero-enteric anastomotic stricture) and type of urinary derivation. Sogni et al.^18^ stated that peri- and postoperative complication rates between patients undergoing RC with IC or ON are comparable, however the latter had a longer postoperative hospital stay than the others. Furthermore, readmission after RC remains common. Skolarus et al.^25^ identified about 25.5% of 1782 patients who underwent a RC, that were readmitted within 30 days. They found no difference in length of stay between age group or type of UD. Stimson et al.^26^ found that gender and age adjusted Charlson Comorbidity Index (CCI) were independent predictors of readmission, while type of UD was no predictive of readmission. Analyzing specific complications after RC, van Hemelrijck et al.^27^ identified 7608 patients who underwent radical cystectomy from 1968 to 2004 and required hospitalization following radical cystectomy. Patients with ON had higher rate of urinary infections and septicemias than the ones who received IC. In 2015 Wuethrich et al.^20^ included a consecutive series of 224 elderly patients who underwent RC with different urinary diversions (including ON). The median age was 79.2 and the 90-days complication rate was 54.3% in the ON group, 56.7% in the IC group, and 63.6% in the UCS group. The highest complication rate occurred in the first 30 days and was lower in patients with ON (35 patients). Whitin 90 days post-operative there was no difference in the incidence of complications between UD. The most common complications observed were gastrointestinal (15%), or infections and cardiac injury (13%). The mortality rate at 90 days was 0% in patients with ON, 13% in IC and 10% in UCS group. In 2020 Demaegd et al.^28^ included 604 patients who underwent RC for BC with different UD (445 IC and 159 ON). The short-term complication rate was comparable in both types of UD and depended on male gender, age adjusted Charlson comorbidity index (aCCI) ⩾3, and American Society of Anesthesiologists (ASA) score ⩾3. On the other hand, patients with ON had a higher long-term complication rate regardless of age, gender, ASA or CCI.^29^^30^

## Quality of life

The diagnosis of MIBC and the consequent therapeutic choice expose the elderly patient to a series of possible complications that can affect the quality of life. Lately a lot of emphasis has been given to this aspect and a lot of QoL data is being reported. In 2007 a study by Saika et al.^29^ evaluated the Qol in the elderly patients (75> yo) who underwent RC. About 109 patients were evaluated after a urinary derivation (UCS, IC or ON) and it was noted that there were no substantial differences in terms of Qol, although many patients with ON were disappointed with their urinary derivation compared to their pre-operative expectations. In a study by Sogni et al.^18^ 32 of 105 patients undergoing RC completed specific questionnaires on Qol, emerging no difference between IC or ON. In a further study by Dutta et al.^30^ patients (23 with IC and 49 with ON) completed 2 Qol evaluation surveys, the RAND 36-Item Health Survey and the Functional Assestment of Cancer Therapy (FACT-G); no differences emerged in QoL between the two groups on the former survey (RAND), while in the latter one (FACT-G) emerged a better score in the areas of functional and emotional well-being for patients with ON. Another study analyzed the role of another procedure-specific QoL metric, named FACT-vanderbit Cystectomy Index (VCI). This one included the FACT-G and other 17 additional questions. About 190 patients were evaluated, and patients with IC scored an higher health-related QoL compared with ON; also in this case the preoperative expectations were disappointed by the functional outcomes, impacting on the QoL.^31^ Lavdaniti and Zyga^32^ in their review analyzed the QoL using EORTC QLQ-C30 and FACT-BL questionnaire in patients undergoing RC with urinary diversion (IC or ON): there were no statistically significant difference on QoL parameters between ON and IC patients, demonstrating that each type of derivation had advantages and disadvantages, depending on the particular case. Siddiqui and Izawa,^33^ for example, argued that IC is the best choice for elderly people with acceptable levels of urinary diversion. In contrary Sogni et al. suggested that ON could be suitable for elderly with good scores in the questionnaires for QoL after RC. In a recent multicentric cohort study by Tostivint et al.^34^ 73 of 162 patients undergoing RC with only ON completed specific questionnaires: the European Organization for Research and Treatment of Cancer (EORTC) generic (QLQ-C30) and BCa-specific instruments (QLQ-BLM30), the Urinary Symptoms Profile (USP), and a voiding diary during three-day. Among these patients median age was 64 years old (58–68) and patients had a high global QoL at long-term follow up (from 20th months to 54th months).^35^

## Conclusion

RC remains one of the most complex surgery and exposes the patients to a high risk of complications. Many studies have shown that BC mainly affects older people and therefore the therapeutic decision are often complicated. Many studies demonstrated clinical and surgical outcomes in octogenarians undergoing CR regardless of the type of UD. A further problem is the definition of “Elderly” which can vary in each single study. The most common cut-off used is 65 years. BC mostly affect old patients, with 71.4% of new diagnoses in over 65 years and the incidence peaking at age 85. The type of UD was at the center of the review, in elderly patients it was preferred to opt for a non-continent derivation in the majority of cases, but in literature there are no evidence about this preference and it may represent a patient-selection bias. However, the data analyzed have highlighted that offering an orthotopic urinary derivation is often appropriate and age should not be considered as an absolute criterion of exclusion for an ON reconstruction. Age must be considered in the set of functional abilities, health state and QoL of the patient.
